# Metabolic Alterations Associated With Right Ventricular Dysfunction in Pulmonary Arterial Hypertension: The Modulatory Effects and Improvement Mechanisms of Exercise

**DOI:** 10.31083/RCM37460

**Published:** 2025-07-30

**Authors:** Sitong Chen, Gengmin Liang, lokfai Cheang, Qiang Qu, Xinli Li

**Affiliations:** ^1^State Key Laboratory for Innovation and Transformation of *Luobing* Theory, Department of Cardiology, The First Affiliated Hospital With Nanjing Medical University, 210029 Nanjing, Jiansgu, China

**Keywords:** pulmonary arterial hypertension, right ventricular, metabolism, exercise training

## Abstract

Pulmonary arterial hypertension (PAH) is characterized by a significant increase in pulmonary arterial pressure, leading to right ventricular failure (RVF), limited exercise capacity, and increased mortality risk. Right ventricular function is a critical determinant of exercise capacity and prognosis in patients with PAH. Meanwhile, alterations in cellular metabolism and bioenergy are common features in PAH, with the differential regulation of metabolic pathways playing a significant role in right ventricular dysfunction (RVD). Mitochondria, essential organelles responsible for energy production, biosynthetic pathways, and signal transduction, are particularly implicated in differential regulation. Exercise is increasingly recognized as a beneficial adjunct therapy; however, specific recommendations are often lacking in official guidelines. This review examines the changes in metabolic pathways associated with RVD in PAH, including glycolysis, glucose oxidation, fatty acid oxidation, glutamine metabolism, and arginine metabolism. Furthermore, this article discusses how exercise can modulate the aforementioned metabolic pathways to improve metabolic disturbances in the right ventricle and enhance right heart function. These are essential for developing effective rehabilitation strategies.

## 1. Introduction

Pulmonary arterial hypertension (PAH) [1, 2] is the first category in the World Symposium on Pulmonary Hypertension (WSPH) classification, with an incidence of approximately 48 to 55 cases per million adults. PAH primarily affects the small pulmonary arteries, leading to adverse vascular remodeling, progressive increases in pulmonary vascular pressure, right ventricular failure (RVF), and ultimately premature mortality. Despite significant improvements in patient prognosis due to targeted pharmacological therapies, exercise limitation remains a prominent feature in PAH patients [3], and is associated with adverse outcomes in this population [4, 5]. The ability of right ventricle (RV) to adapt or compensate for the stress of pulmonary hypertension is a critical factor influencing exercise capacity and outcomes in pulmonary vascular disease [6]. Changes in cellular metabolism and bioenergy are common features of PAH patients. Cellular metabolism includes pathways such as glycolysis and glucose oxidation, fatty acid metabolism, glutamine metabolism, arginine metabolism, etc. Mitochondria are important organelles in cellular metabolism. The heart relies entirely on mitochondrial oxidative phosphorylation (OXPHOS) to generate adenosine triphosphate (ATP) for energy supply. An increasing body of evidence suggests that patients with right ventricular dysfunction (RVD) exhibit various cellular metabolic abnormalities in PAH, including impaired mitochondrial oxidative capacity, reduced cardiac efficiency, and altered substrate utilization patterns [7, 8, 9], such as increased glycolysis and glutamine utilization, increased glutamine utilization, and reduced fatty acid β-oxidation. Studies have shown that exercise training can improve RV function by enhancing the metabolism of RV cardiomyocytes. The European Cardiology Society/European Respiratory Society (ECS/ERS) has classified specialized low-dose exercise training as a Class Ⅱa B recommendation [5]. However, there are no specific guidelines or recommendations regarding the types or intensities of exercise for patients. This article primarily reviews the relationship between RV metabolism and exercise in PAH patients. We explore the changes in metabolic pathways during RVD, and then explain how scientifically designed aerobic exercise can improve cardiac function through metabolic pathway modulation.

## 2. Unique Anatomy and Physiology of the Right Ventricle

RV connects the systemic venous return and the pulmonary vascular bed [10]. Both ventricles pump similar amounts of blood, but the RV works against much lower resistance since pulmonary vascular resistance is approximately one-third of systemic vascular resistance. What is different is the coronary arteries of RV deliver blood continuously during both heart contraction and relaxation. This means when pulmonary artery pressure reaches or surpasses systemic aortic pressure, it may lead to ischemic injury of the RV myocardium. Compared with rest state, the RV during exercise compensates for heightened oxygen demand mainly by extracting more oxygen from the blood rather than coronary vasodilation.

## 3. Pathophysiology of Right Ventricular Dysfunction in PAH

RV decompensation is the leading cause of mortality among patients with PAH [11]. Persistent elevation of afterload induces adaptive remodeling of RV (Table 1). This compensatory mechanism leads to the development of myocardial hypertrophy. However, RV hypertrophy rarely achieves complete compensation. Increased wall stress and relative decreased capillary density can lead to RV ischemia and then result in RVF [12]. Furthermore, reduced coronary perfusion in PAH patients exacerbates RV ischemia. Studies have shown that the severity of RV ischemia and reduced right coronary artery flow correlate with RV mass and end-diastolic pressure [13].

**Table 1.** **Structural and functional changes in RVD in PAH**.

Change type	Specific change	Mechanism	Clinical implications
Structural	RV dilation	Increased afterload	Reduced cardiac output
Structural	RV hypertrophy	Compensatory response	Increased wall stress
Functional	Reduced RV EF	Myocardial stiffness	Decreased stroke volume
Functional	Impaired RV contractility	Oxidative stress and inflammation	Dyspnea, fatigue
Hemodynamic	Increased RV pressure	Pulmonary vascular resistance	RV-PA uncoupling

RVD, right ventricular dysfunction; PAH, pulmonary arterial hypertension; RV, right ventricular; EF, ejection fractions; RV-PA, right ventricular-pulmonary arterial.

The pathophysiological processes that initiate or promote RVF include myocyte hypertrophy, fibrosis, ischemia, neurohormonal activation, inflammation, and metabolic substrate shifts [14]. This section primarily describes the alterations in metabolic pathways associated with RVD in PAH patients.

## 4. Metabolic Alterations in Right Ventricular Dysfunction

The heart is one of the most metabolically active organs in the body, relying on efficient mitochondrial OXPHOS to generate ATP. Cardiac myocytes contain a high density of mitochondria [7]. Glucose and fatty acids are the primary sources of ATP production in the heart. In healthy adults, the heart primarily uses fatty acid oxidation (FAO) for energy production, generating 40–70% of its ATP through this pathway. By comparison, glucose oxidation provides a smaller but still significant contribution, producing about 20–30% of cardiac ATP [15]. The energy metabolism pathways in cardiomyocytes also include glutamine metabolism, arginine metabolism, redox reactions, one-carbon metabolism, as well as the tricarboxylic acid (TCA) cycle and the electron transport chain (ETC) [7].

Studies have identified metabolic abnormalities in PAH patients and animal models [16, 17, 18]. As PAH progresses, the RV undergoes significant metabolic changes, including increased reliance on glycolysis, greater utilization of glutamine, and decreased β-oxidation of fatty acids (Table 2). This metabolic shift likely results from progressive RV hypertrophy in PAH, creating a relatively oxygen-deficient environment within cardiomyocytes [14]. While this metabolic adaptation initially improves ATP production efficiency in cardiomyocytes, it ultimately contributes to pathological heart remodeling. This process worsens both the relaxation and contraction capabilities of RV [19]. These changes decrease the heart’s overall efficiency and consequently diminishes the exercise capacity of patients to some extent.

**Table 2.** **Key metabolic alterations in RVD of PAH**.

Metabolic pathways	Parameters
Glycolysis and glucose oxidation	↑ ^18^FDG uptake
	↑ Glycolytic gene, e.g., hexokinase (*HK*)*2* and solute carrier family 2 member 3 (*SLC2A3*)
	↓ Oxygen consumption
	↓ ^14^C-glucose oxidation
	HIF-α presence in nuclei of cardiomyocytes
Fatty acid oxidation	↓ FAO
	↓ Long-chain acylcarnitine
	↑ Expression of key enzyme, e.g., carnitine palmitoyltransferase 1 (CPT1)
	↑ Fatty acid metabolites by metabolomics
	↑ Lipid content, Ceramide, Triglycerides
Glutaminolysis	↑ Glutamine transporter solute carrier family 1 member 5 (*SLC1A5*)↑ Glutamine
	↑ ^14^C-glutamine metabolism
Arginine metabolism	↑ Serum arginase activity
	↓ NO bioavailability

^18^FDG, ^18^Fluorodeoxyglucose; FAO, fatty acid oxidation; NO, nitric oxide; HIF, hypoxia-inducible factor. ↑, indicates increase; ↓, indicates reduction.

### 4.1 Glycolysis and Glucose Oxidation

In patients with PAH, pathological changes in both the hypertrophied RV and remodeled pulmonary vessels disrupt normal glucose metabolism, characterized by increased glycolysis alongside decreased oxidation. This pattern resembles the “Warburg effect” observed in cancer cells [20, 21, 22]. Fluorodeoxyglucose positron emission tomography (FDG-PET) can quantitatively assess the uptake of ^18^F-fluorodeoxyglucose (FDG) in the heart. Clinical studies using FDG-PET imaging have demonstrated significantly increased FDG uptake in both cardiac and pulmonary tissues of PAH patients compared to healthy controls. The elevated FDG uptake is inversely correlated with RV function [23, 24]. As FDG uptake increases, the levels and activities of glycolysis-related enzymes in the hearts of PAH patients also rise. Researchers have observed an increase in the key glycolytic enzyme hexokinase (HK) and upregulation of the gene solute carrier family 2 member 3 (*SLC2A3*), which encodes for glucose transporter (GLUT)3 [25].

Moreover, myocardial hypoxia can activate hypoxia-inducible factor 1-alpha (HIF-1α). Studies have demonstrated that HIF-1α is essential for the development of PAH [26]. HIF-1α can mediate the transcriptional upregulation of pyruvate dehydrogenase kinase (PDK). Activated PDK then leads to a reduction in pyruvate dehydrogenase (PDH), inhibiting OXPHOS pathways. Additionally, HIF-1α enhances glucose transporter expression and redirects pyruvate away from mitochondria, creating additional inhibition of glucose oxidation pathways. This results in insufficient production of water and oxygen, leading to a hypoxic state in the cells. Consequently, this exacerbates systemic hypoxia and promotes the development and progression of PAH.

### 4.2 Fatty Acid Oxidation

FAO is the primary pathway for oxygen consumption, requiring 12% more oxygen than glucose oxidation to produce an equivalent amount of ATP. In cardiomyocytes, fatty acids are converted into acylcarnitine, which is then transported into mitochondria for ATP production. Studies have shown that levels of acylcarnitine in the RV of PAH patients are lower [27], indicating that FAO is inhibited in PAH. Dysregulation of fatty acid metabolism can lead to the toxic accumulation of lipid substances. Research has found that the key enzyme for fatty acid metabolism, carnitine palmitoyltransferase 1 (CPT1), is upregulated in PAH, and its overexpression promotes the transport of fatty acids into the mitochondria. Hemnes *et al*. [28] demonstrated significant accumulation of toxic lipid intermediates—such as ceramides, triglycerides, and diacylglycerols—within right ventricular mitochondria. This accumulation was particularly prominent in both hereditary PAH cases with bone morphogenetic protein receptor type 2 (BMPR2) mutations, and experimental BMPR2-deficient mouse models. This lipid overload disrupts mitochondrial function, triggers cardiomyocyte apoptosis, and contributes to RVD and RVF [27, 29]. A plasma metabolomics analysis in PAH patients revealed that sphingolipid metabolic pathways are associated with RV dilation and N-terminal pro-brain natriuretic peptide (NT-proBNP) levels [8].

### 4.3 Glutaminolysis

Glutamine metabolism and the Warburg effect are common metabolic pathways in cancer [30] and PAH, enabling cells to grow rapidly. In PAH patients, RV exhibit upregulated glutamine metabolism, associated with microvascular rarefaction and ischemia in the RV [31]. In the monocrotaline (MCT)-induced rat model, the expression of the gene solute carrier family 1 member 5 (*SLC1A5*), which mediates glutamine uptake, is upregulated [32]. Ischemia is a significant pathophysiological mechanism that promotes RVF. Glutamine metabolism and its derived metabolites play a crucial role in the maladaptive remodeling of the RV in PAH.

### 4.4 Arginine Metabolism

Arginine is a semi-essential amino acid and serves as a substrate for nitric oxide synthase (NOS) and arginase (ARG). In the pulmonary vasculature, endothelial nitric oxide synthase (eNOS) is the primary NOS isoform. It converts arginine into nitric oxide (NO) and citrulline. NO is a powerful vasodilator that reduces pulmonary vascular resistance and RV afterload [33]. Arginine can also be metabolized by ARG to produce ornithine and urea. Studies have shown that due to elevated serum ARG activity, PAH patients have much lower plasma levels of arginine, citrulline, and the arginine-to-ornithine ratio [22, 34]. On one hand, this increased activity competes with NOS for arginine, limiting NO production. On the other hand, the accumulation of the metabolic product ornithine can be converted to glutamate and α-ketoglutarate (αKG). These substances feed into the TCA cycle and affect cellular metabolism and mitochondrial bioenergetics. In animal models of PAH, inhibition of ARG has been shown to reduce right ventricular systolic pressure (RVSP), decrease pulmonary tissue remodeling, and enhance NO bioavailability [35].

## 5. Exercise and Right Ventricular Function in PAH

Cardiac reserve refers to the ability of cardiac output to increase in response to metabolic demands. It includes stroke volume reserve and heart rate reserve. Healthy individuals can maintain cardiac reserve well by increasing heart rate and stroke volume during exercise [36]. But cardiac reserve is diminished in PAH patients, resulting in reduced cardiac output response during exercise. And then symptoms such as dyspnea, fatigue, and congestion appear. The exercise capacity and exercise reserve of patients with PAH are closely related to RV function [6]. Echocardiographic findings in PAH patients show clear patterns linked to exercise limitation. These patients typically demonstrate enlarged right atrial (RA) and RV areas, along with a higher eccentricity index. The findings also reveal reduced heart function, seen through lower fractional area change (FAC) and decreased tricuspid annular plane systolic excursion (TAPSE) [9]. RVD, particularly the decline in RV systolic function and RV-PA uncoupling, is a key factor limiting maximum cardiac output and exercise capacity [37]. Furthermore, exercise reserve has been shown to correlate more closely with RV afterload and ventricular stiffness [38].

Besides medicines, exercise training also plays an important role in treating PAH patients. The European Society of Cardiology (ESC)/European Respiratory Society (ERS) guidelines for PH classify specialized low-dose exercise training as a Class IIa recommendation [5]. In mouse models of MCT-induced pulmonary hypertension [39], combined aerobic and resistance training can prevent increases in pulmonary vascular resistance, inhibit RV and pulmonary structural remodeling, and reduce oxidative stress. Several studies have indicated that low-intensity exercise training can effectively improve exercise capacity, enhance quality of life, reduce hospitalizations, and potentially improve hemodynamics in PAH patients [40, 41, 42]. Exercise can protect the heart and reduce cardiovascular risk factors and events by decreasing myocardial oxidative stress, promoting physiological cardiac hypertrophy, inducing angiogenesis, and facilitating adaptive changes in cardiac metabolism [43]. This section focuses on how exercise induces cardiac metabolic adaptations, improves RV metabolic dysregulation, inhibits RV remodeling, and enhances symptoms and prognosis in PAH patients.

## 6. Metabolic Adaptations Induced by Exercise

Exercise training enhances cardiac workload and myocardial oxygen consumption. This leads to significant metabolic changes in the heart. The rate of ATP production in the myocardium increases, accompanied by elevated catabolism of carbohydrates and fatty acids. As a result of increased lactate and free fatty acid levels during exercise training, glucose uptake and glycolysis are comparatively diminished [44]. Additionally, the heightened concentrations of lactate and free fatty acids facilitate the absorption and utilization of fatty acids [45]. Exercise promotes metabolic substrate shift toward fatty acid utilization, improving cardiac metabolic flexibility to some extent. Thereby, exercise enhances energy production efficiency and myocardial energy supply.

Mitochondria are essential organelles within cells, responsible for energy production and storage, as well as participating in various cellular metabolism and signaling processes. During exercise, mitochondrial dynamics—including fusion, fission, and autophagy—are induced to maintain homeostasis and ensure a steady supply of metabolic energy [46, 47]. Furthermore, exercise can enhance mitochondrial biogenesis by activating peroxisome proliferator-activated receptor gamma coactivator 1-alpha (PGC-1α) and eNOS [48]. These alterations in mitochondrial function and quality can ultimately improve the ability of myocardial cells to undergo glucose oxidation and FAO.

The shear forces generated during exercise can lead to an increase of Ca^2^⁺ levels in cytoplasm and mitochondria, which play a crucial role in various cellular processes. Ca^2^⁺ can promote ATP production through enhanced ATPase activity, dehydrogenase activity, and nicotinamide adenine dinucleotide phosphate (NADH) oxidation [49]. The elevated intracellular Ca^2^⁺ interacts with calmodulin to phosphorylate, leading to the phosphorylation and activation of eNOS, and subsequently promoting NO production [50]. eNOS is expressed in coronary endothelial cells and cardiomyocytes.

In cardiomyocytes, eNOS catalyzes the conversion of arginine to NO and is involved in regulating mitochondrial respiratory function and electron transport. Exercise can enhance the binding of eNOS with the mitochondrial membrane [51]. Research has shown that exercise can restore arginine levels, helping to alleviate the substrate utilization limitation of eNOS and increasing NO production [8]. NO can further inhibit the interaction between reactive oxygen species (ROS) and Ca^2^⁺ in mitochondria, thereby protecting cardiomyocytes [52].

**Fig. 1.**
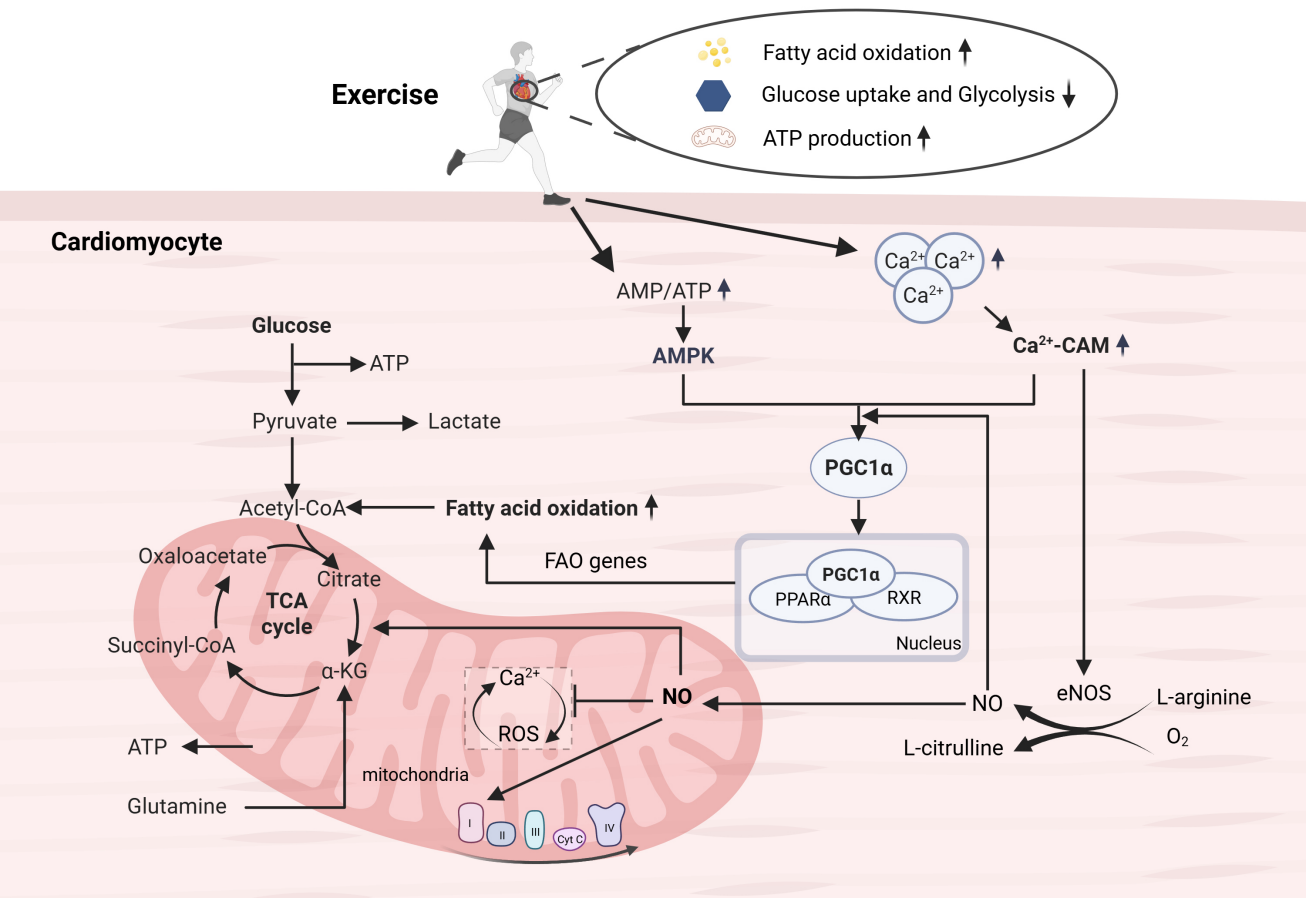
**Metabolic adaptations induced by exercise**. Exercise can promote the conversion of metabolic substrates to fatty acids, and enhance mitochondrial oxidative phosphorylation capacity by promoting mitochondrial production and maintaining internal homeostasis, thereby improving metabolic and energy production efficiency. The PPAR, PGC-1α, and AMPK signaling pathways play important roles in these processes. ATP, adenosine triphosphate; NO, nitric oxide; ROS, reactive oxygen species; Ca^2^^+^-CAM, Ca^2^^+^-Calmodulin; TCA, tricarboxylic acid; FAO, fatty acid oxidation; FAO genes, FATP1, CPTI, MCAD, etc. AMPK, AMP-activated protein kinase; PPAR-α proliferator-activated receptor alpha; RXR, retinoid X receptor; *FATP 1*, 1fatty acid transport protein 1; CPTI, carnitine palmi-toyltransferase I; MCAD, medium-chain acyl-CoA dehydrogenase. This figure is created in https://www.biorender.com/.

The peroxisome proliferator-activated receptor (PPAR), PGC-1α, and AMP-activated protein kinase (AMPK) signaling pathways have been demonstrated to regulate the expression of genes involved in FAO glycolysis, and mitochondrial biogenesis during exercise. The AMP/ATP ratio increases during exercise, activating the AMPK signaling pathway [53]. All of the above factors, including increased production of NO, enhanced interaction of Ca^2^^+^-Calmodulin kinase, and activation of AMPK, can lead to the upregulation and activation of PGC-1α in cardiomyocytes [54, 55]. PGC-1α can form a transcriptional regulatory complex with peroxisome proliferator-activated receptor alpha (PPAR-α) and retinoid X receptor (RXR) [56], promoting the downstream transcription of genes involved in FAO pathways, such as fatty acid transport protein 1 ( *FATP 1*), carnitine palmitoyltransferase I (CPTI), and medium-chain acyl-CoA dehydrogenase (MCAD). These gene regulations improve FAO while reducing the toxic accumulation of free fatty acids in the myocardium. Additionally, PGC-1α can stimulate mitochondrial biogenesis [57, 58] (Fig. 1).

## 7. Clinical Implications and Future Directions

While exercise is increasingly recognized as a beneficial adjunct therapy, its role is still evolving, and specific recommendations are often lacking in official guidelines. Exercise can be effectively integrated into the treatment regimen for PAH patients to improve RV function and enhance overall quality of life, with supervised aerobic and resistance exercises showing promising results in increasing exercise capacity and reducing RV afterload. Selecting appropriate patients for exercise programs requires careful evaluation of baseline functional status, hemodynamic parameters, and comorbidities, with exercise prescriptions starting at low intensity and gradually increasing as tolerated, guided by cardiopulmonary exercise testing (CPET).

However, implementing exercise programs presents challenges such as the need for specialized monitoring, potential exacerbation of symptoms, and patient compliance, which are compounded by the heterogeneity of PAH and its progression. Addressing these issues requires a multidisciplinary approach and close collaboration between healthcare providers and patients. Future research should focus on elucidating the specific metabolic and functional benefits of exercise in PAH, including the underlying mechanisms and long-term outcomes, through randomized controlled trials to establish optimal exercise protocols for different patient subgroups. Additionally, studies should explore the role of metabolic and genetic biomarkers in predicting exercise response and the potential of exercise to complement or reduce the need for pharmacological interventions.

## 8. Conclusion

RV plays a crucial role in PAH. This article reviews the metabolic disturbances associated with RVD in PAH, including abnormalities in glycolysis, increased glutamine utilization, and decreased β-oxidation of fatty acids. Exercise can improve myocardial metabolism by promoting metabolic substrate shift toward fatty acid utilization and enhancing mitochondrial OXPHOS capacity. These adaptations improve metabolic efficiency and energy production. This provides a theoretical foundation for developing scientific, effective and personalized exercise therapy strategies. However, clinical implementation of exercise rehabilitation in PAH remains limited, particularly due to the lack of standardized exercise guidelines. Future research could focus on changes in myocardial metabolic biomarkers before and after exercise, and explore the optimal exercise regimen for different subgroups of PAH patients through randomized controlled trials. Additionally, studies should examine the temporal dynamics of cardiac metabolic adaptation following exercise training, including both acute responses and chronic adaptations. The long-term cardioprotective effects of exercise in PAH patients also warrant further investigation.
